# Identifying heterogeneity in the Hawthorne effect on hand hygiene observation: a cohort study of overtly and covertly observed results

**DOI:** 10.1186/s12879-018-3292-5

**Published:** 2018-08-06

**Authors:** Kuan-Sheng Wu, Susan Shin-Jung Lee, Jui-Kuang Chen, Yao-Shen Chen, Hung-Chin Tsai, Yueh-Ju Chen, Yu-Hsiu Huang, Huey-Shyan Lin

**Affiliations:** 1 Division of Infectious Diseases, Department of Internal Medicine, Kaohsiung Veterans General Hospital, Kaohsiung, Taiwan; 2 Faculty of Medicine, School of Medicine, National Yang-Ming University, Taipei, Taiwan; 3 Department of Health-Business Administration, Fooyin University, Kaohsiung, Taiwan; 4 Graduate Institute of Environmental Education, National Kaohsiung Normal University, Kaohsiung, Taiwan; 5 Infection Control Unit, Kaohsiung Veterans General Hospital, Kaohsiung, Taiwan

**Keywords:** Hand hygiene, The Hawthorne effect, Overt observation, Covert observation, Hand hygiene compliance

## Abstract

**Background:**

Observation and feedback are core strategies of hand hygiene (HH) improvement. Direct overt observation is currently the gold standard method. Observation bias, also known as the Hawthorne effect, is a major disadvantage of this method. Our aim was to examine the variation of the Hawthorne effect on HH observation in different healthcare groups and settings.

**Methods:**

A prospective cohort study was performed in a tertiary teaching hospital during a 15-month period. Up to 38 overt observers (82% nurses) and 93 covert observers (81% medical students) participated in HH observation. The HH events observed overtly were matched for occupation, department, observation time, and location with those observed covertly. The data of matched pairs were then analysed to detect possible Hawthorne effects on different variables.

**Results:**

A total of 31,522 HH opportunities were observed (4581 overtly, 26,941 covertly). There were 3047 matched pairs after 1:1 matching of overt and covert observations. The overall HH compliance was higher with overt observation than with covert observation (78% vs. 55%, *p* < 0.001). The Hawthorne effect was nearly three times larger in nurses (30 percentage points) than in physicians (11 percentage points) and was significantly greater in outpatient clinics (41 percentage points) than in intensive care units (11 percentage points). The magnitude of the Hawthorne effect varied among healthcare worker occupations and observation locations (*p* values both < 0.001) but not among departments, observation times, or HH indications.

**Conclusions:**

Heterogeneity in the Hawthorne effect may influence the interpretation of overt observations and prevent the correct identification of target populations with poor HH compliance. Therefore, directly observed HH compliance may not be an adequate performance indicator for infection control.

## Background

Hand hygiene (HH) is an effective measure to prevent healthcare-associated infection [1–4]. Observation and feedback make up one of the five main strategies to improve HH [3]. Direct observation remains the gold standard method of evaluating HH compliance [3, 4]. However, direct observation has several limitations, including the ability to observe only a small percentage of HH opportunities in real-world scenarios, the need for a large number of observers and a considerable amount of time, the lack of a standardized training/validation process, and observation bias [5, 6].

Observation bias, also known as the Hawthorne effect, plays a critical role in evaluating HH compliance. When the Hawthorne effect was estimated by the difference between direct overt and covert observation results, the magnitude of the effect on HH compliance ranged from 7 to 16 percentage points (PPs) before 2009 [7–9] to 30–34 PPs after Five Moments for Hand Hygiene became a standard in 2009 [10, 11]. Srigley et al. even noted a surprising 3-fold increase in HH compliance under direct observation vs under an electronic monitoring system (EMS) [12]. Hagel et al. reported a similar outcome by using electronic handrub dispensers to record HH events and captured a 2.6-fold higher HH density when healthcare workers (HCWs) were under observation than when they were not (21 vs 8 HH events per hour) [13]. McLaws and Kwok demonstrated direct observation rates were inflated by an average of 55–64 PPs (2.8–3.1 times higher) than automated surveillance rates by an EMS in a medical ward [14]. The Hawthorne effect may alter HCWs’ usual behaviour and often leads to overestimation of HH compliance [5, 6, 9, 15]. At least three methods have been proposed to help avoid or decrease the Hawthorne effect besides the use of EMS. These include 1) letting HCWs habituate to the presence of observers, although there is still no standard method for clinical application [3, 6]; 2) using indirect methods to monitor HH compliance, such as monitoring consumption of alcohol handrubs [15]; and 3) covertly observing HH compliance [3, 15].

Few studies have provided a detailed description and subgroup analysis of the Hawthorne effect [9, 11]. Kohli et al. conducted an observational study comparing the observation results of three infection control members and one student intern in three inpatient care units and found that the Hawthorne effect was more pronounced in high-performing than low-performing units. The authors speculated that the staff in high-performing units take pride in their work and may wish to show their good performance to recognized observers [9]. Kovacs-Litman and colleagues described a much lower difference between covertly and overtly observed compliance rates in physicians than in nurses (19 PPs vs 41 PPs, *p* < 0.0001) [11]. However, the two studies had several study design flaws, including the presence of selection bias [9, 11], a small number of observers [9], only HH opportunities occurred before and after contact with the patient or patient’s environment being observed [9], and a relatively small number of HH opportunities [9]. Since healthcare facilities around the world spend so much time and utilize so many people for HH compliance observation (mostly overt), further research to investigate the Hawthorne effect is necessary [6].

The main purpose of the present study is to explore the heterogeneity of the Hawthorne effect in different HCW subgroups and settings. We analysed covert and overt observation data in the same hospital during the same period after controlling for observation place, time, department, and occupation category. The study results may clarify the impact of the Hawthorne effect under various HH circumstances.

## Methods

### Settings

All HH opportunities in the study were observed in Kaohsiung Veterans General Hospital (KVGH) from October 2012 through December 2013. Located in Kaohsiung City, Taiwan, KVGH is a tertiary teaching hospital with 1408 beds and more than 3600 HCWs. KVGH has regarded HH as a core element of infection control for nearly two decades and has implemented HH improvement strategies according to WHO guidelines soon after their issuance in 2009. The HH promotion campaign led to a significant decrease in the rate of healthcare-associated infection – from 3.7 to 3.1% (*p* = 0.002) – and reductions in overall healthcare cost and average hospital stay [16]. Direct overt observation and feedback have been routinely performed in KVGH since 2009 as described in the WHO technique manual for observers. The overall rate of HH compliance in 2011 was 73% [16].

The HH compliance of HCWs was both overtly and covertly observed in wards, intensive care units, and outpatient clinics. The study protocol was approved by the KVGH Institutional Review Board (VGHKS13-CT4–05). All covert observers signed informed consent forms. Overt observers were exempted from signing informed consent forms because overt observation of HH compliance has long been a routine infection control practice in this hospital.

### Overt observers

Overt observers were recruited among HCWs at KVGH. HCWs who were interested in becoming HH compliance observers could register to receive training. A total of 38 overt observers were included in the study after receiving training and certification. Those 38 observers were assigned to specific areas in the hospital and asked to observe for at least one hour every month. The observers could freely decide their observation dates and times. The minimum length of each observation period was 20 min. The hospital administration periodically and publicly commended the overt observers for their service but provided no monetary compensation.

### Covert observers

The covert observers were medical students who participated in a HH training project in 2012–2013. The project was part of a campaign to improve HH in medical students after we found that doctors had the lowest HH compliance of all HCWs. The project recruited 149 students, who, similar to their overt observer counterparts, completed the same training and underwent the same validity check. In all, 93 of these students became covert observers. Their observer duties were performed at the sites of their internship rotations in teaching hospitals across Taiwan. A previous study described detailed observation results in hospitals other than KVGH [17]. In this study, only covert observations in KVGH were used for analysis.

### Training and certification

Overt and covert observers received nearly identical HH instruction from the same instructors. The course comprised lectures on HH knowledge, handrubbing and handwashing techniques, observation skills, video watching, and group discussion. The main teaching materials were the guidelines, video, and technical reference manual issued by the WHO [3, 18, 19]. After receiving the training, the observers were certified by passing a written test and demonstrating observer proficiency in five health care scenarios filmed by the infection control team. The process of HH observation via video watching also served as the means to reach inter-observer agreement. Those observers who were correct ≥80% of the time were considered qualified. Arrangements were made for both overt and covert observers to join discussion meetings every 3 months after being qualified. At those meetings, the concepts of HH observation will be reinforced, and problems about observation will be discussed to reach a consensus.

### Covert observation techniques

The only difference between the courses taught to overt and covert observers was that covert observers were also taught techniques of covert observation. We designed a unique coding system to aid participants in remembering the observation results correctly and to reduce recall bias [17]. The numbers 1–5 were used to represent 5 HH indications, along with R for handrubbing, W for handwashing, and N for no HH action. For example, when a physician washed his or her hands with handrub between visiting 2 consecutive patients, observers would quietly memorize 14R. The observers were encouraged to record the codes as soon as possible after observing no more than 2–3 HH opportunities.

### Observation and reporting

The locations of overt observation were assigned by the infection control team of KVGH, and the overt observers decided their observation times and durations. The covert observers were encouraged to monitor compliance during their daily routines, such as ward rounds, in order not to interfere with their internship learning or be detected by other HCWs. The observation data records were uploaded to a website that could be accessed only by qualified observers and KVGH infection control team members. The covert observers were compensated $0.30 for every HH opportunity observed and reported. Overt observers received no monetary rewards.

### Statistical analysis

HH opportunities observed in the study period were analysed according to different variables using descriptive statistics. Thereafter, the overt and covert observations were matched for patient department, HCW occupation, observation location, and observation time using a 1:1 exposure and non-exposure matching method. We used the difference in the rate of HH compliance between overt and covert observers in these matched pairs as a measure of the Hawthorne effect. McNemar’s tests were used to analyse the differences between overt and covert observer data in each category. Generalized estimating equations were then used to test for differences between overt and covert observer data among categories within each variable. For example, for the variable of occupation, McNemar’s test was used to assess the differences in the compliance rates of overt and covert observation for nurses, physicians, caregivers and other HCWs, respectively; generalized estimating equations were performed to test for differences between overt and covert observer data among physicians, nurses, caregivers, and other HCWs.

Only matched pairs with the same number of HH indications regardless of observation method were included in the analysis for the variable “number of indications per hand hygiene opportunity”. Only the matched pairs with the same HH indication regardless of observation method were used to analyse the variable “hand hygiene indication”. All analyses were performed using SPSS 22.0 for Windows (IBM, Armonk, NY).

## Results

### Overall hand hygiene compliance

A total of 131 observers participated in the study from October 2012 through December 2013, including 38 overt observers and 93 covert observers. The overt observers were mainly nurses (82%), followed by infection control team members (8%), physicians (5%), and research assistants (5%). The covert observers were medical students (81%) as well as students in the departments of physical therapy (15%), Chinese medicine (2%), and dentistry (1%).

During the study period, 31,522 HH opportunities were observed, including 4581 overtly observed opportunities and 26,941 covertly observed opportunities. Before matching, the overall HH compliance rate was 81% by overt observation and 59% by covert observation.

### Matched analysis results

There were 3047 matched pairs after matching. Nurses were the most common HCW category represented in the matched pairs (69%). Most HH actions occurred in the ward (67%) and during the day (81%). The rate of overall HH compliance was higher by overt observation than by covert observation (78% vs. 55%, *p* < 0.001), with a difference of 24 PPs. The higher rate of HH compliance by overt observation than by covert observation (*p* < 0.001) was also true for most hospital departments, HCW occupations, observation locations, and observation times but not among caregivers (*p* = 0.30) or other HCWs (*p* = 0.18). Table 1 describes the results for the 3047 matched pairs in detail.

**Table 1:** The difference in hand hygiene compliance in 3047 pairs of overtly and covertly observed hand hygiene opportunities stratified by department, occupation, observation time, and location

Overt observation	Number of matched pairs (%) Covert observation		Matched odds ratio	*p* value^a^
	Hand rubbing/washing	No hand rubbing/washing		
Department				
Medicine (*N* = 1212)			3.0	< 0.001
Hand rubbing/washing	510 (42.1)	418 (34.5)		
No hand rubbing/washing	139 (11.5)	145 (12.0)		
Surgery (*N* = 1258)			3.6	< 0.001
Hand rubbing/washing	475 (37.8)	482 (38.3)		
No hand rubbing/washing	135 (10.7)	166 (13.2)		
Paediatrics (*N* = 463)			2.7	< 0.001
Hand rubbing/washing	292 (63.1)	112 (24.2)		
No hand rubbing/washing	41 (8.9)	18 (3.9)		
Gynaecology/obstetrics (*N* = 114)			2.9	0.001
Hand rubbing/washing	60 (52.6)	35 (30.7)		
No hand rubbing/washing	12 (10.5)	7 (6.1)		
Occupation				
Nurse (*N* = 2105)			5.3	< 0.001
Hand rubbing/washing	994 (47.2)	778 (37.0)		
No hand rubbing/washing	146 (6.9)	187 (8.9)		
Physician (*N* = 619)			1.6	< 0.001
Hand rubbing/washing	241 (38.9)	179 (28.9)		
No hand rubbing/washing	114 (18.4)	85 (13.7)		
Caregiver (*N* = 106)			1.4	0.302
Hand rubbing/washing	26 (24.5)	27 (25.5)		
No hand rubbing/washing	19 (17.9)	34 (32.1)		
Others (*N* = 217)			1.3	0.184
Hand rubbing/washing	76 (35.0)	63 (29.0)		
No hand rubbing/washing	48 (22.1)	30 (13.8)		
Time^b^				
Day shift (*N* = 2493)			3.1	< 0.001
Hand rubbing/washing	1120 (44.9)	838 (33.6)		
No hand rubbing/washing	273 (11.0)	262 (10.5)		
Early night shift (*N* = 516)			3.7	< 0.001
Hand rubbing/washing	208 (40.3)	190 (36.8)		
No hand rubbing/washing	52 (10.1)	66 (12.8)		
Late night shift (*N* = 38)			9.5	< 0.001
Hand rubbing/washing	9 (23.7)	19 (50.0)		
No hand rubbing/washing	2 (5.3)	8 (21.1)		
Location				
Ward (*N* = 2034)			3.7	< 0.001
Hand rubbing/washing	815 (40.1)	774 (38.1)		
No hand rubbing/washing	208 (10.2)	237 (11.7)		
Intensive care unit (*N* = 880)			1.9	< 0.001
Hand rubbing/washing	498 (56.6)	211 (24.0)		
No hand rubbing/washing	111 (12.6)	60 (6.8)		
Outpatient department (*N* = 133)			7.8	< 0.001
Hand rubbing/washing	24 (18.0)	62 (46.6)		
No hand rubbing/washing	8 (6.0)	39 (29.3)		
Number of indications per hand hygiene opportunity				
1 (*N* = 2301)			3.1	< 0.001
Hand rubbing/washing	963 (41.9)	801 (34.8)		
No hand rubbing/washing	260 (11.3)	277 (12.0)		
2 (*N* = 42)			2.7	0.227
Hand rubbing/washing	28 (66.7)	8 (19.0)		
No hand rubbing/washing	3 (7.1)	3 (7.1)		
Hand hygiene indication				
Before touching a patient (*N* = 270)			3.1	< 0.001
Hand rubbing/washing	96 (35.6)	99 (36.7)		
No hand rubbing/washing	32 (11.9)	43 (15.9)		
Before clean/aseptic procedures (*N* = 19)				0.063
Hand rubbing/washing	10 (52.6)	5 (26.3)		
No hand rubbing/washing	0 (0)	4 (21.1)		
After body fluid exposure risk (*N* = 18)			6.0	0.125
Hand rubbing/washing	8 (44.4)	6 (33.3)		
No hand rubbing/washing	1 (5.6)	3 (16.7)		
After touching a patient (*N* = 287)			3.1	< 0.001
Hand rubbing/washing	140 (48.8)	99 (34.5)		
No hand rubbing/washing	32 (11.1)	16 (5.6)		
After touching patient surroundings (*N* = 56)			1.1	1.000
Hand rubbing/washing	16 (28.6)	15 (26.8)		
No hand rubbing/washing	14 (25.0)	11 (19.6)		
Overall (*N* = 3047)			3.2	< 0.001
Hand rubbing/washing	1337 (43.9)	1047 (34.4)		
No hand rubbing/washing	327 (10.7)	336 (11.0)		

^a^McNemar’s tests were used to analyse the differences between overtly and covertly observed hand hygiene compliance in each category

^b^Day shift, 0800–1600 h; early night shift, 1600–2400 h; late night shift, 2400–0800 h

### Differences between overt and covert observation results

The difference between overt and covert observation results was approximately three times larger in nurses than in physicians (30 PPs vs. 11 PPs) and nearly four times larger in outpatient clinics than in intensive care units (41 PPs vs 11 PPs). Further analysis revealed that the differences between overt and covert observation results were different among various HCW occupations and observation locations (*p* values both < 0.001) but not among various departments (*p* > 0.05) (Table 2).

**Table 2:** Differences in the Hawthorne effect among various departments, occupations, observation times, observation locations, number of indications per hand hygiene opportunity, and hand hygiene indications

Variable	Pair no.	Hand hygiene compliance (%)		Hawthorne effect (percentage point difference)	*p* value^a^
		Overt observation	Covert observation		
Department (*N* = 6094)					0.255
Medicine	1212	76.6	53.3	23.3	
Surgery	1258	76.1	48.5	27.6	
Others	577	86.5	70.2	16.3	
Occupation (*N* = 6094)					< 0.001
Nurse	2105	84.2	54.2	30.0	
Physician	619	67.9	57.4	10.5	
Caregiver	106	50.0	42.5	7.5	
Others	217	64.1	57.1	7.0	
Time^b^ (*N* = 6094)					1.000
Day shift	2493	78.5	55.9	22.6	
Early night shift	516	77.1	50.4	26.7	
Late night shift	38	73.7	28.9	44.8	
Location (*N* = 6094)					< 0.001
Ward	2034	78.1	50.3	27.8	
Intensive care unit	880	80.6	69.2	11.4	
Outpatient department	133	64.7	24.1	40.6	
Numbers of indications per hand hygiene opportunity^c^ (*N* = 4686)					0.541
1	2301	76.7	53.2	23.5	
2	42	85.7	73.8	11.9	
Hand hygiene indication^d^ (*N* = 1300)					0.130
Before touching a patient	270	72.2	47.4	24.8	
Before clean/aseptic procedures	19	78.9	52.6	26.3	
After body fluid exposure risk	18	77.8	50.0	27.8	
After touching a patient	287	83.3	59.9	23.4	
After touching patient surroundings	56	55.4	53.6	1.8	
Overall (*N* = 6094)	3047	78.2	54.6	23.6	

^a^Generalized estimating equations were used to test whether there were significant differences in the Hawthorne effect among different categories of each variable

^b^The day shift was 0800–1600 h, the early night shift 1600–2400 h, and the late night shift 2400–0800 h

^c^Only the matched pairs with the same number of indications for both overt and covert observation methods were included

^d^Only the matched pairs with the same HH indication for both overt and covert observation methods were included

## Discussion

This study comprehensively investigated the extent of the Hawthorne effect on HH compliance observation and revealed that the Hawthorne effect differed significantly for HCW occupations and observation locations but not for departments, observation times, or HH indications. In this study, several attempts were made to reduce potential bias. In order to minimize observer bias, both overt and covert observers received the same training and underwent the same certification process; moreover, a large group of observers were recruited to observe a massive number of HH opportunities. To minimize selection bias, we used matched analysis to balance 4 important variables between the overt and covert observation groups. The overall Hawthorne effect was comparable between our study (24 PPs) and previous studies of direct HH compliance observation (7–34 PPs) [7–11]. The study results may influence how we interpret direct observer audits of HH compliance in the future.

The heterogeneity of the Hawthorne effect on HH observation has several impacts on HH monitoring. First, Hawthorne effect heterogeneity should be taken into consideration when identifying risk factors for not washing one’s hands. The identification of risk factors for HH noncompliance was often based on analyses of overt observation data in previous studies [20, 21]. For example, HH compliance was considered lower in physicians than in nurses [7, 20]. Is this proposition true, or do the observations reflect a greater Hawthorne effect in nurses than physicians [11]? The answer may even vary among health care facilities. Second, some experts may take advantage of the Hawthorne effect on HH observation to improve HH compliance, that is, using the strategy of frequent HH observation to stimulate HCWs to wash their hands more often. Implementing the strategy in the scenario with a higher Hawthorne effect will maximize the efficiency of HH improvement programmes. Third, using HH compliance by overt observation as a performance indicator to compare different HCW occupations, wards, and hospitals is not adequate. The major purpose of overt observation should remain the provision of feedback to HCWs.

Our finding that the Hawthorne effect impacted physicians less than nurses (11 PPs vs. 30 PPs) was comparable to the finding of Kovacs-Litman and colleagues (19 PPs vs. 41 PPs) [11]. Our study did not show the previously reported more pronounced Hawthorne effect on HH in higher-performance units and lack of a significant Hawthorne effect on HH in low-performance units [9]. Kwok et al. noted that a socially cohesive ward is more likely than a socially isolated ward to adopt an intervention for HH improvement [22]. This difference may partially explain why units with high HH performance, which are presumed to be more social cohesive, have a larger response to overt observation, and vice versa. However, in our study, HH compliance was higher and the Hawthorne effect on compliance was lower in intensive care units than in wards or the outpatient department; moreover, HH compliance was lowest and the Hawthorne effect was highest in the surgical department. We consider the association between HH performance and the Hawthorne effect to be undetermined. Further studies are needed to address this issue.

The use of EMS to monitor HH compliance has attracted increasing attention in recent years [12–14, 23, 24]. An interesting point is that the estimated Hawthorne effect is often larger when HH compliance from overt observation is compared to EMS measures (1.6 to 3.1 times higher) [12–14] than to covertly observed measures (7–34 PPs or 12–69% higher) [7–11], including this study (24 PPs or 43% higher). This difference may imply that the Hawthorne effect can only be reduced, rather than eliminated, by covert observation. As long as the HCWs are not alone, whether the persons nearby tend to observe HH or not, the Hawthorne effect exists. To go one step further, the EMS may also produce a Hawthorne effect to a certain extent if the HCWs are aware of the existence of EMS. The differences in results between various observation methods may cause another form of Hawthorne effect heterogeneity.

There are several limitations to the study. A major limitation is that the Hawthorne effect was measured by calculating the difference between overt and covert observation results, as in previous studies [7–9, 11]. Although the Hawthorne effect may contribute the most to the difference, selection and observer bias will certainly exist despite statistical correction. For example, the covert observers may tend to observe HH at busier times to see more HH opportunities in less time due to the monetary incentive. Busier times were associated with lower HH compliance; thus, a form of selection bias may be present. In addition, the professions of the observers in overt and covert observation were considerably different, and we did not analyse the impact of observer profession (e.g., nurse vs physician observers) on the Hawthorne effect in the study. Observer bias may occur as a result. We believe that an accurate measurement of the Hawthorne effect can be obtained only through well-designed controlled trials, for example, the same group of observers conducting both overt and covert observations in designated areas during the same period of time. Until perfect results are available, the results retrieved from a large cohort with careful stratification and matching may provide useful information. Second, this investigation is a single-centre study that was conducted in a hospital with good HH implementation. The impact of the Hawthorne effect may vary from hospital to hospital, and we recommend caution in applying the study results to other hospitals. Third, covert observation may not be generally acceptable in other hospitals or countries, which would prevent the generalization of the study method.

## Conclusions

In this study, we demonstrated not only the existence of the Hawthorne effect but also a significant difference in the Hawthorne effect among different HCW occupations and observation locations. Due to the heterogeneity of the Hawthorne effect, overt observation should not be used as the sole method of comparing performance among different clinical settings, HCWs, or hospitals. Overt observation continues to be the main method of HH observation because it is easy to perform and provides more information for feedback than EMS provides. However, better methods of benchmarking performance are needed.

## Abbreviations

EMS: Electronic monitoring system
HCW: Healthcare worker
HH: Hand hygiene
KVGH: Kaohsiung Veterans General Hospital
PP: Percentage point
WHO: World Health Organization
